# Chemical risk and safety awareness, perception, and practices among research laboratories workers in Italy

**DOI:** 10.1186/s12995-020-00268-x

**Published:** 2020-06-16

**Authors:** Rosa Papadopoli, Carmelo Giuseppe Angelo Nobile, Alessandro Trovato, Claudia Pileggi, Maria Pavia

**Affiliations:** 1 Department of Health Sciences, Medical School, University of Catanzaro “Magna Græcia”, Via T. Campanella, 115, 88100 Catanzaro, Italy; 2 Department of Pharmacy, Health and Nutritional Science, University of Calabria, Arcavacata di Rende, 87036 Cosenza, Italy; 3 Department of Experimental Medicine, University of Campania “Luigi Vanvitelli”, Via L. Armanni, 5, 80138 Naples, Italy; 4 Department of Health Sciences, University of Catanzaro “Magna Græcia”, Via T. Campanella, 115, 88100 Catanzaro, Italy

**Keywords:** Occupational exposure, Chemical hazards, Chemical risk, Safe handling, Toxic, Mutagenic, Cancerogenic

## Abstract

**Introduction:**

Exposure to chemical compounds occur**s** in numerous occupational settings, among which the research and healthcare laboratories have not been adequately investigated. These settings are characterized by an extreme variability of the used compounds and by the frequent turnover of young researchers. The main objectives of the study were to explore the occupational exposure to hazardous chemical substances among research laboratory workers; to assess their awareness and perceptions regarding chemical hazards; to investigate adherence to guidelines on safe handling of chemical compounds; and to analyze the effects of several factors on these outcomes of interest.

**Methods:**

The survey was conducted among research laboratories workers who were exposed to chemical substances during their activity. Subjects completed a questionnaire exploring knowledge, attitudes and behaviors related to chemical hazards involved in research activities.

**Results:**

Enrolled subjects were 237, for an 81.7% response rate. More than 90 hazardous chemical substances were used in the surveyed laboratories. A correct knowledge on hazardous chemicals was significantly more likely in younger researchers, in those manipulating a higher number of hazardous chemicals, and in those with a higher number of years of training in the attended laboratory; 54.4% of the workers said they felt very exposed to chemical risk. Correct practices in the laboratories were significantly more likely in researchers who perceived to have a low exposure to chemicals, but a high exposure to biological risk, who agreed with the statement that colleagues handle chemicals following safety procedures and who perceived to have received an adequate training in the management of accidents and first aid.

**Conclusions:**

Our results showed significant gaps in knowledge and scarce preparedness in the adherence to safety processes to prevent and contain risks related to use of chemical compounds in research laboratories.

## Introduction

There is evidence that the professional exposure to chemical, physical and biological risks may cause severe health effects and the European Agency for Safety and Health at work reported that in 2015 17% of workers in the European Union (EU) declared to be exposed to chemical products or substances for at least a quarter of their working time [1].

To guarantee protection from the risk that can be posed by chemicals in consumer products, at the workplace or in the environment, the Italian government has adopted the European Regulations concerning the Registration, Evaluation, Authorization and Restriction of Chemicals (REACH) [2] and the Classification, Labelling and Packaging (CLP) of substances and mixtures [3]. Moreover, EU legislation has been included in a comprehensive act that regulates all actions involved in workers’ health safety and protection (Decree 81/2008) [4].

Studies have associated workplace exposures to hazardous chemical substances with both acute and mild effects such as skin rashes, or eye irritation or burns, as well as severe effects, such as adverse reproductive outcomes (including infertility, spontaneous abortions, and congenital malformations), and possibly leukemia and other cancers [5–7]. The health risks of chemicals depend on several factors, including the intrinsic characteristics of substances, as well as the extent and the duration of exposure [8].

Exposure to chemical compounds occurs in numerous occupational settings, among which the research and healthcare laboratories, that are characterized by the extreme variability of the used compounds and by the frequent turnover of young researchers. However, chemical hazards have been explored in detail in several industrial and agricultural occupational settings [9–12], whereas in research or healthcare laboratories, attention has been particularly devoted to biological hazards [13–15]. Indeed, in a review on chemical exposure and related health risks in laboratory workers, studies (mainly retrospective cohorts and case-control) investigating incidence and mortality for several cancers, including numerous cancer sites, and occurrence of reproductive adverse events, such as miscarriage, low birth weight and malformations in chemistry and biology laboratory workers have been extensively reviewed, and they report contrasting results, with some showing an association and others not [16]. Investigation in this field could provide useful information to assess the extent of use and exposure to hazardous chemical compounds, and to verify how much researchers are able to protect themselves from chemical risks.

Therefore the main objectives sought by this survey were the following: 1) to explore the occupational exposure to chemical substances classified as toxic, mutagenic and cancerogenic among research laboratory workers; 2) to assess workers’ awareness and perceptions regarding chemical hazards involved in research activities; 3) to investigate adherence to guidelines on safe handling of chemical compounds; and 4) to analyze the effects of several factors on these outcomes of interest.

## Methods

### Study design

The survey was conducted between January and March 2019 among all research laboratory workers in two Universities of Southern Italy. All subjects (associate and assistant professors, research fellows, Ph.D, graduate and undergraduate students, and technologists) attending all laboratories, who were exposed to chemical substances (toxic, mutagenic and cancerogenic) during their activity, were considered eligible, therefore no sampling was performed**.** All eligible subjects who consented to participate received an hard copy of an anonymous questionnaire including an explanatory opening paragraph reporting the purpose of the study, advising that there was no obligation to complete the questionnaire, and reassuring that the information obtained would remain confidential. The return of the completed questionnaire was considered as written consent to participation.

### Survey instruments

The questionnaire was developed based on the literature review of comparable studies [17–20]. The first version was submitted to a small representative group of workers to ensure question clarity, format and sequence, and the information collected through the pilot study was not included in the final analysis. The final questionnaire consisted of 49 items distributed into the following six sections: the first one included socio-demographic and professional characteristics (gender, age, education level, marital and employment status, time in practice with chemical exposure, previous professional practice in other laboratories); the second section explored the health status, particularly chronic health conditions (diabetes, chronic respiratory diseases, cardiovascular diseases, cephalalgia, chronic renal or liver failure, etc.), smoking status, eventual drugs consumption, and the recent health history. The third section investigated knowledge on the hazardous chemicals (toxic, mutagenic and cancerogenic) used in research laboratories; the fourth assessed attitudes and perceptions towards use of chemicals; the fifth explored the practice with chemicals during research activities in the laboratories; finally participants were asked to indicate their preferred sources of information about the chemical risks factors and their effects as related to their occupational activity, eventual attendance to training courses on laboratory safety and specifically on chemical hazards as well as their perceived need of additional information on chemical hazards.

Moreover, the safety data sheet (SDS), completed by each exposed worker according to the REACH [2], was reviewed to collect data on chemical substances (toxic, mutagenic and cancerogenic) to which they were exposed during their activity. Specifically, the information regarding the chemicals consisted of name, modes of handling and storing, classification according to CLP (hazard and precautionary phrases) [3], physical and chemical properties, quantities and duration of use.

After having taken into account the characteristics of exposure (duration and handling modes)**,** all the substances listed in the SDS were classified, according to CLP Regulation, as Category 1 (cancerogenic and mutagenic to humans), Category 2 (lethal if in contact with systems of the human body), Category 3 (toxic) and Category 4 (irritant). For substances included in more than one category, the higher risk category was attributed.

### Statistical analysis

Statistical analysis was developed using STATA software program, version 16 (Stata Corporation. College Station, Tx).

Data were summarized using frequencies and percentages for categorical data and mean and standard deviations for continuous data.

Univariate and stepwise multivariate logistic regression analyses were performed to determine the independent association of explanatory variables with the following outcomes of interest: knowledge on the hazardous chemicals used by research laboratory workers (Model 1); protective activities adopted by the workers during their research activities in the laboratories (Model 2). In Model 1, to measure “good knowledge” the cut-off was set at having correctly answered to at least 75% of questions on knowledge, therefore the workers were divided in those who reported at least 9 correct answers out of the 12 questions in the knowledge section of the questionnaire, versus all others; in Model 2 the workers were divided into those who reported to check correct operation of closed system when handling chemicals, stated that they do not eat in lab and that they wash hands before and after lab procedures, versus all others. Stepwise multivariate logistic regression models were developed according to the Hosmer and Lemeshow strategy [21], and independent variables for which *p* value was 0.25 or less at the univariate analysis, were included in the models. Furthermore, socio-demographic variables and those that, regardless of the results of univariate analysis, were judged to potentially have influence on the outcomes of interest were also included in the appropriate model. Therefore, the following independent variables were included in both models: sex, age, employment status, laboratory site, number of hazardous chemicals used and number of months working in the attended lab.

Moreover, in Model 2 we also included visits to general practitioner (GP) in the previous year, the “good knowledge” variable, variables that investigated perception on safety of workplace, risk associated to chemical, biological and radiation exposure, perception that colleagues handle chemicals following safety procedures, that proper personal protective equipment (PPE) are available in the laboratory, that safety measures protect from unwanted effects related to exposure to chemicals, that exposure to cancerogenic chemicals is extremely low, perceptions of adequate training on management of accidents, on decontamination procedures in case of accidental spillage of hazardous chemicals, on use of PPE, on interpretation of safety data sheets, and the perception that inadequate training on safe handling of chemicals can contribute to risk of injury. An additional file shows the list of variables and the related categories [see Additional file 1]. Adjusted odds ratio (OR) and 95% confidence intervals (CI) were calculated.

The study protocol was ratified by the Regional Ethical Committee (ID N.25/2019/01/17).

## Results

### Subjects characteristics

A total of 290 eligible subjects were asked to participate in the study. Two hundred thirty-seven of them returned the completed questionnaire, for an 81.7% response rate. The majority of the workers was composed by females (77.6%), mean age was 35.9 (range 21–64) years, 42.3% of participants were married/cohabitant and 80.6% declared to have never smoked. More than half of the responders were Ph.D. students and research fellows, and 25.5% had been working in the current lab for less than 1 year; overall, more than half reported at least one working experience in other labs. About half of the workers reported to have a chronic illness and 67.9% had attended a GP in the previous year (Table 1).

**Table 1:** Demographic, professional and health status characteristics of the responders

Characteristic	N	%	Mean ± SD
Gender **(237)**^a^			
Female	184	77.6	
Male	53	22.4	
Age, years **(237)**^a^			35.9 ± 9.8
≤ 30	85	35.9	
31–40	87	36.7	
> 40	65	27.4	
Marital status **(213)**^a^			
Single/separated/divorced	123	57.7	
Married/cohabitant	90	42.3	
Smoking status **(237)**^a^			
Never smoker	191	80.6	
Current smoker	30	12.7	
Past-smoker	16	6.7	
Employment status **(236)**^a^			
Temporary workers	119	50.4	
Permanent workers	117	49.6	
Laboratory site **(237)**^a^			
University of Catanzaro	157	66.2	
University of Cosenza	80	33.8	
Number of months working in the attended lab **(235)**^a^			74.4 ± 82
≤ 24	92	39.1	
25–48	46	19.6	
49–120	42	17.9	
> 120	55	23.4	
Number of hazardous chemicals used in the attended lab **(237)**^a^			
≤ 5	97	46.0	6.6 ± 3.3
6–10	109	46.0	
> 10	31	13.1	
Working experience in other labs **(237)**^a^			
Yes	127	53.6	
No	110	46.4	
Chronic health conditions (**237**)^ab^			
*No*	118	49.8	
Yes	119	50.2	
*Allergies*	*71*	*59.7*	
*Cephalalgia*	*52*	*43.7*	
*Dysmetabolic diseases*	*15*	*12.6*	
*Cardiovascular diseases*	*4*	*3.4*	
*Respiratory diseases*	*4*	*3.4*	
*Other*	*14*	*11.8*	
Visits to GP in the previous year **(237)**^a^			
Yes	161	67.9	
No	76	32.1	

*GP* general practitioner

^a^Number of responders to the questions

^**b**^Percentages do not add up to 100 due to multiple responses

### Knowledge on chemical hazards and on safety in the laboratories

Table 2 reports the results on workers’ level of knowledge about hazardous chemicals (toxic, mutagenic and cancerogenic) used in the laboratories. Only 50.2% knew that reference legislation on hazardous chemicals is shared by different countries. When assessing knowledge on ways of contamination with chemicals, the majority (69.2%) of respondents was aware that chemicals in oil more likely penetrate skin than chemicals in water, 74.7% correctly reported that hand washing does not promote the absorption of chemicals from skin into the body and 81.9% knew that contamination with chemicals does not occur exclusively through the inhalation or dermal absorption. Only 48.5% was aware that there is a threshold dose for non-genotoxic carcinogens and that the exposure to levels below this value does not represent a risk for developing cancer. The vast majority correctly reported that acrylamide can affect health by dermal absorption (80.6%), inhalation (80.2%) and ingestion (94.1%); moreover, 73.8% of respondents was aware that formaldehyde has cancerogenic effects. Knowledge on PPE resulted inadequate, considering that 61.2% wrongly believed that all types of gloves in the laboratory are classified as PPE. Conversely, 86.9% correctly identified the meaning of an indicated pictogram, and 60.3% correctly indicated that the H statement in safety data sheets identifies hazards related to the use of chemicals.

**Table 2:** Respondents’ knowledge on chemical hazards

	N (%) Strongly agree or Agree	N (%) Uncertain	N (%) Strongly disagree or Disagree
**Statements**			
Reference legislation on hazardous chemicals is independently identified in different countries	65 (27.4)	53 (22.4)	**119 (50.2)**
Chemicals in oil more likely penetrate skin than chemicals in water	**164 (69.2)**	35 (14.8)	38 (16)
Hand washing promotes the absorption of chemicals from skin into the body	17 (7.2)	43 (18.1)	**177 (74.7)**
There is a threshold dose for non-genotoxic carcinogens below which they do not induce neoplasms	**115 (48.5)**	62 (26.2)	60 (25.3)
Acrylamide can affect health if:			
a. You touch it	**191 (80.6)**	21 (8.9)	25 (10.5)
b. You breathe in air that contains it	**190 (80.2)**	17 (7.2)	30 (12.6)
c. You eat it	**223 (94.1)**	11 (4.6)	3 (1.3)
Formaldehyde is an hazardous chemical but does not have cancerogenic effects	39 (16.5)	23 (9.7)	**175 (73.8)**
All types of gloves in the laboratory are classified as personal protective equipment (PPE)	145 (61.2)	28 (11.8)	**64 (27)**
The only ways of contamination with chemicals are inhalation and dermal absorption	34 (14.3)	9 (3.8)	**194 (81.9)**
The following pictogram indicates a flammable substance:	19 (8)	12 (5.1)	**206 (86.9)**
The H statement in safety data sheets identifies the hazards relating to use of the chemicals	**143 (60.3)**	67 (28.3)	27 (11.4)

Note: The correct answers are in bold

An overall “good knowledge” on hazardous chemicals, indicated by those who correctly answered to at least 9 out of the 12 questions on knowledge, was reported by less than half of laboratory researchers (46%) and, at the multivariate analysis, this correct knowledge was significantly more likely in younger researchers, in those manipulating a higher number of hazardous chemicals, in those with a higher number of years of training in the attended laboratory, and was significantly different in the two selected Universities (Table 3).

**Table 3:** Determinants of knowledge, and professional practice concerning hazardous chemicals in research laboratories

Variable			Univariate analysis			Multivariate analysis		
***Outcome: Knowledge on hazardous chemicals used by research laboratory workers***	**N**	**%**	**OR**	**95% CI**	**p**	**OR**	**95% CI**	**p**
*Log-likelihood = − 144.245, χ*^*2*^ *= 34.5, P value = 0.0001, No. of obs. = 234*								
Laboratory site								
University of Catanzaro	85	54.1	1.00*			1.00*		
University of Cosenza	24	30	0.36	0.02–0.64	0.001	0.27	0.12–0.58	0.001
Gender								
Male	28	52.8	1.00*			1.00*		
Female	81	44	0.7	0.38–1.29	0.258	0.54	0.27–1.07	0.080
Age, years								
≤ 30	40	47.1	1.00*			1.00*		
31–40	45	51.7	1.20	0.66–2.19	0.541	0.49	0.21–1.14	0.102
> 40	24	36.9	0.65	0.34–1.27	0.215	0.25	0.08–0.76	0.015
Employment status								
Temporary workers	54	45.4	1.00*			1.00*		
Permanent workers	54	46.2	0.98	0.77–1.26	0.922	2.1	0.85–5.16	0.105
Number of hazardous chemicals used in the attended lab								
≤ 5	42	43.3	1.00*			1.00*		
6–10	58	53.2	1.48	0.85–2.58	0.156	2.56	1.34–4.91	0.004
> 10	9	29	0.53	0.22–1.28	0.161	2.20	0.71–6.75	0.167
Number of months working in the attended lab								
< 24	39	42.4	1.00*			1.00*		
24–48	23	50	1.35	0.66–2.76	0.398	1.38	0.65–2.92	0.391
49–120	28	66.7	2.71	1.26–5.83	0.010	2.99	1.31–6.85	0.009
> 120	19	34.6	0.71	0.35–1.43	0.347	Backward elimination		
***Outcome: Self-reported preventive measures adopted by the workers during their research practices in the laboratories***	**N**	**%**	**OR**	**95% CI**	**p**	**OR**	**95% CI**	**p**
*Log-likelihood = − 118.733, χ*^*2*^ *= 76.16, P value < 0.0001, No. of obs. = 234*								
Number of hazardous chemical used in current lab								
≤ 5	36	37.1	1.00*			1.00*		
6–10	48	44	1.33	0.76–2.33	0.313	1.48	0.76–2.89	0.247
> 10	9	29	0.69	0.28–1.66	0.413	Backward elimination		
Number of months working in the attended lab								
< 24	29	31.5	1.00*			1.00*		
24–48	24	52.2	2.36	1.14–4.90	0.020	3.24	1.33–7.89	0.009
9–120	19	45.2	1.79	0.84–3.79	0.127	2.02	0.82–4.94	0.122
> 120	20	36.4	1.24	0.61–2.50	0.547	Backward elimination		
Visit to GP in the previous year								
No	25	32.9	1.00*			1.00*		
Yes	68	42.2	1.49	0.84–2.64	0.17	1.41	0.69–2.86	0.338
Perception of safety of workplace								
Unsafe	14	27.5	1.00*			1.00*		
Somewhat safe	30	33.3	1.32	0.62–2.81	0.469	1.69	0.62–4.59	0.299
Safe	49	51	2.75	1.32–5.73	0.007	1.75	0.64–4.79	0.274
Perception of risk associated to chemical exposure								
< 4 (not much)	51	47.2	1.00*			1.00*		
≥ 4 (much)	42	32.6	0.53	0.31–0.91	0.02	0.4	0.18–0.86	0.020
Perception of exposure to biological risk								
1–2 (not exposed)	31	33	1.00*			1.00*		
3 (moderately exposed)	19	38.8	1.28	0.62–2.63	0.491	2.35	0.92–5.96	0.072
4–5 (very exposed)	43	45.7	1.71	0.94–3.09	0.074	2.81	1.24–6.38	0.013
Risk perception of radiation exposure								
1–2 (not exposed)	66	37.1	1.00*			1.00*		
3 (moderately exposed)	20	50	1.69	0.85–3.38	0.133	2.53	0.97–6.56	0.055
4–5 (very exposed)	7	36.8	0.98	0.37–2.63	0.984	2.22	0.64–7.65	0.205
The laboratory colleagues handle chemicals following safety procedures								
1–2 (strongly disagree or disagree)	10	19.2	1.00*			1.00*		
3 (uncertain)	18	40.9	2.9	1.16–7.25	0.022	7.24	2.16–24.22	0.001
4–5 (strongly agree or agree)	65	46.1	3.59	1.67–7.71	0.001	2.97	1.08–8.15	0.034
Availability of proper PPE								
1–2 (strongly disagree or disagree)	14	26.9	1.00*			1.00*		
3 (uncertain)	11	27.5	1.02	0.40–2.59	0.951	0.48	0.18–1.27	0.140
4–5 (strongly agree or agree)	68	46.9	2.39	1.19–4.79	0.014	Backward elimination		
Perception that safety measures protect from unwanted effects related to exposure to chemicals								
1–2 (strongly disagree or disagree)	24	30	1.00*			1.00*		
3 (uncertain)	7	23.3	0.71	0.26–1.87	0.490	0.2	0.058–0.7	0.012
4–5 (strongly agree or agree)	62	48.8	2.22	1.23–4.02	0.008	Backward elimination		
Perception that exposure to cancerogenic chemicals is extremely low								
1–2 (strongly disagree or disagree)	47	38.2	1.00*			1.00*		
3 (uncertain)	7	22.6	0.47	0.18–1.18	0.108	0.26	0.08–0.81	0.020
4–5 (strongly agree or agree)	39	47	1.43	0.81–2.51	0.211	Backward elimination		
Perception of having received an adequate training in management of accidents and first aid								
1–2 (inadequate)	11	19.3	1.00*			1.00*		
3 (just adequate)	33	43.4	3.2	1.44–7.13	0.004	3.93	1.5–10.26	0.005
4–5 (totally adequate)	49	47.1	3.72	1.73–7.98	0.001	2.27	0.89–5.81	0.086
Perception of having received an adequate training in the interpretation of safety data sheets								
1–2 (inadequate)	12	26.1	1.00*			1.00*		
3 (just adequate)	13	24.1	0.89	0.36–2.22	0.817	0.17	0.06–0.45	< 0.001
4–5 (totally adequate)	68	49.6	2.79	1.33–5.84	0.006	Backward elimination		

*GP* general practitioner

* Reference category

The following variables were removed from model 2 by backward elimination procedure: gender; laboratory site; employment status; knowledge on hazardous chemicals; perception that inadequate training in safe chemicals handling can contribute to risk of injury; training regarding management of accidents; training regarding use of PPE; training regarding decontamination procedures in case of accidental spillage of hazardous chemicals

### Attitudes and perceptions on chemical hazards and on safety in the laboratories

Table 4 shows workers’ attitudes towards use of hazardous chemicals; when workers were asked to indicate in a 10-point Likert-type scale ranging from 1 for “totally unsafe” to 10 for “totally safe” the safety perception in the workplace, the mean score was just satisfactory (6.5 ± 2.5). Moreover, when factors contributing to risk of injury were investigated, the scores for high workload, inexperience and inadequate training in safe handling of chemicals were 7.4 (±2.2), 8.6 (±1.8) and 8.6 (±1.7), respectively.

**Table 4:** Respondents’ attitudes towards use of hazardous chemicals

ATTITUDES	N (%)	N (%)	N (%)
	**Unsafe** **(score 1–4)**	**Somewhat safe** **(score 5–7)**	**Safe** **(score 8–10)**
Perception of a safe workplace ^a^	51 (21.5)	90 (38)	96 (40.5)
Factors contributing to risk of injury ^b^:	**Not much** **(score 1–4)**	**Enough** **(score 5–7)**	**Much** **(score 8–10)**
high workload	25 (10.6)	67 (28.2)	145 (61.2)
inexperience	11 (4.6)	34 (14.4)	192 (81)
inadequate training in safe handling of chemicals	6 (2.5)	39 (16.5)	192 (81)
Perception of having received an adequate training regarding ^c^:	**Inadequate** **(score 1–2)**	**Just adequate** **(score 3)**	**Totally adequate** **(score 4–5)**
interpretation of the safety data sheet	46 (19.4)	54 (22.8)	137 (57.8)
management of accidents and first aid	57 (24)	76 (32.1)	104 (43.9)
decontamination procedures in case of accidental spillage of hazardous chemicals	73 (30.8)	82 (34.6)	82 (34.6)
use of PPE	32 (13.5)	64 (27)	141 (59.5)
Perception of exposure to^d^:	**Not exposed** **(score 1–2)**	**Moderately exposed** **(score 3)**	**Very exposed (score 4–5)**
biological risk	94 (39.7)	49 (20.6)	94 (39.7)
chemical risk	38 (16)	70 (29.6)	129 (54.4)
ionizing radiations risk	178 (75.1)	40 (16.9)	19 (8)
environmental risk	136 (57.4)	49 (20.7)	52 (21.9)
work-related stress risk, ergonomic factors risk	73 (30.8)	59 (24.9)	105 (44.3)
Perception of safety related to occupational activity and colleagues:	**Strongly disagree or Disagree**	**Uncertain**	**Strongly agree or Agree**
Exposure to cancerogenic chemicals is extremely low	123 (51.9)	31 (13.1)	83 (35)
Safety measures protect from unwanted effects related to exposure to chemicals	80 (33.7)	30 (12.7)	127 (53.6)
Proper interpretation of label of all hazardous chemicals can prevent laboratory injuries	4 (1.7)	9 (3.8)	224 (94.5)
Proper PPE are available	52 (21.9)	40 (16.9)	145 (61.2)
My laboratory colleagues handle chemicals following safety procedures	52 (21.9)	44 (18.6)	141 (59.5)

^a^ 10-point Likert-type scale ranging from 1 for “totally unsafe” to 10 “totally safe”

^b^10-point Likert-type scale ranging from 1 for “not much” to 10 “very much”

^c^ 5-point Likert-type scale ranging from 1 for “not at all adequate” to 5 for “completely adequate”

^d^ 5-point Likert-type scale ranging from 1 for “not at all exposed” to 5 for “very much exposed”

Only 35% of subjects perceive that exposure to cancerogenic chemicals is extremely low and about half (53.6%) of the workers believe that safety measures prevent from unwanted effects related to exposure to chemicals. The vast majority of respondents (94.5%) believe that a proper interpretation of labels of all hazardous chemicals can protect from laboratory injuries; furthermore, two thirds (61.2%) of the subjects stated that proper PPE are available in the laboratories and that the colleagues in the laboratory handle chemicals following safety procedures (59.5%).

Moreover, a reasonable adequacy of the training regarding interpretation of the safety data sheet, management of accidents and first aid in case of contact with hazardous chemicals, decontamination procedures in case of accidental spillage of hazardous chemicals and use of PPE was reported. In a 5-point Likert-type scale ranging from 1 for “not at all exposed” to 5 for “very much exposed”, 54.4% of the workers said they felt very exposed to chemical risk (score ≥ 4).

### Safety practices with chemical hazards in the laboratories

The review of the 237 SDSs compiled by the workers showed that there were more than 90 hazardous chemical substances used in the surveyed laboratories, with a mean of 6.6 (± 3.3) per subject, and the most frequently reported compounds were methanol, acrylamide, chloroform and formaldehyde, handled by over 50% of the subjects. Workers were exposed to liquid, liquid in solution, solid, powder, and gel substances, mainly to liquids (99.2%) and, according to CLP, 98.3% of respondents reported to be exposed to at least one of the substances classified as “cancerogenic and mutagenic to humans” (Category 1), and simultaneous exposure to several carcinogens was common (87.8%). Moreover, 89.4% of subjects handled chemicals classified as “lethal if in contact with systems of the human body” (Category 2), 15.6% classified as “toxic” (Category 3) and 32.1% classified as “irritant” (Category 4).

Responses on adherence to safe handling guidelines used to minimize exposure showed that 86.5% of respondents check correct operation of closed system when handling chemicals, whereas use of PPE ranged from 99.2% for gloves to 59.1% for protective glasses. More than half (57.4%) stated that they had never eaten in lab, and the remaining that it could have happened occasionally or rarely, when supervision of in progress experiments did not allow to leave the laboratory. About two thirds (69.6%) of the population reported to wash hands before and after lab procedures; furthermore, 53.6% declared to be exposed to chemicals in more than 25% of the working hours.

At the multivariate analysis correct practices in the laboratories were significantly more likely in researchers who had been working in the attended lab for at least 24 months, who perceived to have a low exposure to chemicals, but a high exposure to biological risk, who agreed with the statement that colleagues handle chemicals following safety procedures and who perceived to have received an adequate training in the management of accidents and first aid (Table 3).

### Training on safety and sources of information

Only nearly half (48.9%) of respondents reported that professional training courses on chemical risk were held in their workplace and questions regarding sources of information about occupational chemical hazards and prevention indicated that workers learnt mainly from older colleagues (51.5%), internet (44.3%) and scientific journals (30%). More than three quarters (76.8%) of respondents reported need to improve their knowledge about hazardous chemicals.

## Discussion

To the best of our knowledge, this study represents one of the few attempts to assess knowledge, attitudes and practices regarding chemical hazards and safety in research laboratories. These issues have been investigated in a broad range of laboratory workers, thoroughly assessing the occupational exposure to chemical compounds and the practices involved in the manipulation of these agents.

### Knowledge on chemical hazards and on safety in the laboratories

The results of the study have shown that, although researchers are aware of most investigated chemical hazards and on how they may affect health, they are not very confident on how to protect themselves, since the knowledge on PPE is far from satisfactory. The finding that knowledge was significantly higher in younger researchers and in those with a longer experience in the attended laboratories may be related to the higher probability of having received adequate training courses on laboratory safety. Indeed, training of workers on safety has become compulsory in Italy several years ago [4, 22], but rigorous accomplishment to this rule is more recent; therefore it may be hypothesized that younger researchers have had a higher opportunity to be educated to safety compared to older ones, whereas those who have worked in the laboratory for a longer time, since training courses are held periodically, may have had a higher probability of being engaged in courses on safety as compared to less experienced workers. The finding that researchers who manipulate more chemical compounds have a higher knowledge confirms that experience with chemicals influences the need of an increased awareness related to risks of exposure.

### Perceptions and practices on chemical hazards and on safety in the laboratories

The majority considered their laboratory a safe place, whereas one fifth an unsafe one. This figure is higher compared to the 8% that reported to feel unsafe in a study conducted in academic, government and industry researchers in USA [23]. Reasons perceived to be involved in risk of injury were inexperience and inadequate training in safe handling of chemicals, as well as high workload. This finding deserves careful attention on the opportunity to improve researchers’ self-confidence on the manipulation of chemical compounds, and this is confirmed by the high proportion of respondents who perceive to have not received an adequate training on several aspects of laboratory safety, and particularly on the management of accidents.

We found that almost all subjects reported to handle at least one, but very frequently more than one cancerogenic and mutagenic compound, and a great majority handles “lethal if in contact with systems of the human body” substances. In such a context, it is somewhat alarming that reported safety practices showed that there is still about 30% of researchers that do not wash their hands after manipulating chemical compounds, and that the reported use of PPE is not adequate. It should also be highlighted that, although occasionally, researchers reported to have eaten in the laboratories to supervise experimental processing; this finding is of concern and requires a thoughtful revision of laboratory working shifts.

### Relationship between perceptions and safety practices in the laboratories

The findings of our study on the relationship between perceptions on safety in the laboratories and reported correct practices deserve a thorough attention. According to several Health Behavior Models [24, 25], a higher perception of risk is related to a greater likelihood of behaving safely; therefore, investigation on perceptions of risk is very useful to predict behavior. However, studies investigating the association between perception of risk and related behavior have shown contrasting results and it has been reported that the examination of risk perception in the occupational safety literature has not received the deserved attention and that the nature of the relationship between risk perception and safety behavior remains somewhat unclear. Indeed, in the assessment of perception of risk related to exposure to chemicals, the same hazard may be considered at low risk if all safety organizational and personal precautions are adopted, and at high risk if no safety precautions are adopted; therefore ambiguous statements on risk perception may be related to apparently contradicting results on the association between risk perception and safe behavior. This may have been the case of our study, since we found that correct protection practices in the laboratories were more frequent in researchers who perceive to have a low exposure to chemicals and to have received an adequate training in the management of accidents and first aid, thus suggesting awareness and self-confidence on how to protect themselves from chemical hazards.

### Safety training

Concern is also related to the finding that training courses on the specific topic of chemical risk had not been provided to more than half of participants, and indeed researchers reported that the main sources of information on occupational chemical hazards and related prevention were older colleagues and the internet. The role of older colleagues and particularly of principal investigators and supervisors in laboratories has been object of interest in the investigation of determinants of safe practices, and it has been reported that supervisors and principal investigators’ commitment and monitoring of safety practices motivates and positively affects safety behavior in research laboratories [23, 26]. Therefore, increasing awareness of senior researchers and older colleagues of their role with respect to adherence to safety practices of younger researchers should be promoted. It should also be noted that, although 67.9% of researchers reported to have visited a GP in the previous year, they do not mention physicians as a source of information on occupational chemical hazards and related health effects; this represents a missed opportunity, since it has been reported that GPs have an excellent opportunity to provide information to patients about environmental health concerns [27].

### Strength and limitations

One of the major strengths of this study is related to the specific topic of the survey, that has explored a neglected issue, since very sparse data is available on awareness, perception and practice related to prevention and containment of chemical risks in research laboratories; moreover the study has reached a very high response rate (more than 80%) thus reducing concerns on external validity. Limitations of this study must also be acknowledged. First, the cross-sectional nature of the design allows only to investigate associations between the variables of interest. Moreover, research laboratories were located in universities in Southern Italy, and concerns regarding representativeness and generalizability should be taken into account; however we believe that the sampling methods and the high response rate allow us to be confident on the representativeness of the chosen sample and that our results may be generalized to Italian researchers. Finally, data were self-reported, and no objective assessment of practices in the laboratories was performed, which could have determined a shift of the answers to more correct and safe behaviors. However, this does not appear to be the case in our study, since the findings have also demonstrated poor adherence to prevention practices.

## Conclusions

Our findings showed significant gaps in knowledge and scarce preparedness in the adherence to safety processes to prevent and contain risks related to use of chemical compounds in research laboratories. Occupational training of researchers should be enforced to ensure full awareness of the hazards and of the precautionary measures to prevent or reduce exposures to chemicals, consolidating commitment and monitoring of safety practices of principal investigators and research supervisors.

## Supplementary information

**Additional file 1.** Variables included in the logistic regression models with related categories.

## Data Availability

The datasets used and/or analyzed during the current study are available from the corresponding author on reasonable request.

## Abbreviations

SDS: Safety Data Sheet
REACH: Registration Evaluation Authorization, Restriction of Chemicals
CLP: Classification, Labelling, Packaging
GP: General Practitioner
