# Supplementary material for: Chemical risk and safety awareness, perception, and practices among research laboratories workers in Italy
Source: J Occup Med Toxicol. 2020 Jun 16;15:17. doi: 10.1186/s12995-020-00268-x (PMC7298783; doi:10.1186/s12995-020-00268-x)
Supplement: Supplementary file 1 — Additional file 1. Variables included in the logistic regression models with related categories. [file 12995_2020_268_MOESM1_ESM.docx]

**Additional file 1. Variables included in the logistic regression models with related categories**

| **Variable** | **Code** |
| --- | --- |
| Sex | 0 = male  1 = female |
| Age, in years | 1= ≤ 30  2= 31-40  3= > 40 |
| Employment status | 0 = temporary worker  1 = permanent worker |
| Laboratory site | 0 = University of Catanzaro  1 = University of Cosenza |
| Number of hazardous chemicals used in the attended lab | 1= ≤ 5  2= 6-10  3 = > 10 |
| Number of months working in the attended lab | 1 = < 24  2= 24-48  3 = 49-120  4 = > 120 |
| Visits to general practitioner in the previous year | 0 = no  1 = yes |
| Good knowledge | 0= < 9 correct answers  1 = ≥ 9 correct answers |
| Perception on safety of workplace | 1 = unsafe  2 = somewhat safe  3 = safe |
| Risk associated to chemical exposure | 0 = not much  1 = much |
| Risk associated to biological exposure | 1 = not exposed  2 = moderately exposed  3 = very exposed |
| Risk associated to radiation exposure | 1 = not exposed  2 = moderately exposed  3 = very exposed |
| Perception that colleagues handle chemicals following safety procedures | 1 = strongly disagree or disagree  2 = uncertain  3 = strongly agree or agree |
| Perception that proper PPE are available in the laboratory | 1 = strongly disagree or disagree  1 = uncertain  2 = strongly agree or agree |
| Perception that safety measures protect from unwanted effects related to exposure to chemicals | 0 = strongly disagree or disagree  1 = uncertain  2 = strongly agree or agree |
| Perception that exposure to cancerogenic chemicals is extremely low | 0 = strongly disagree or disagree  1 = uncertain  2 = strongly agree or agree |
| Perception of adequate training on management of accidents | 1 = inadequate  2 = just adequate  3 = totally adequate |
| Perception of adequate training on decontamination procedures in case of accidental spillage of hazardous chemicals | 1 = inadequate  2 = just adequate  3 = totally adequate |
| Perception of adequate training on use of PPE | 1 = inadequate  2 = just adequate  3 = totally adequate |
| Perception of adequate training on interpretation of safety data sheets | 1 = inadequate  2 = just adequate  3 = totally adequate |
| Perception that inadequate training on safe handling of chemicals can contribute to risk of injury | 1 = not much  2 = enough  3 = much |
